# Diverse Functionalization of Aurora-A Kinase at Specified Surface and Buried Sites by Native Chemical Modification

**DOI:** 10.1371/journal.pone.0103935

**Published:** 2014-08-05

**Authors:** Fiona Rowan, Meirion Richards, Marcella Widya, Richard Bayliss, Julian Blagg

**Affiliations:** 1 Cancer Research U.K. Cancer Therapeutics Unit, Division of Cancer Therapeutics, The Institute of Cancer Research, London, United Kingdom; 2 Division of Structural Biology, The Institute of Cancer Research, London, United Kingdom; 3 Proteomics Core Facility, The Institute of Cancer Research, London, United Kingdom; 4 Department of Biochemistry, University of Leicester, Leicester, United Kingdom; Institut de Génétique et Développement de Rennes, France

## Abstract

The ability to obtain a homogeneous sample of protein is invaluable when studying the effect of alterations such as post-translational modifications (PTMs). Selective functionalization of a protein to investigate the effect of PTMs on its structure or activity can be achieved by chemical modification of cysteine residues. We demonstrate here that one such technique, which involves conversion of cysteine to dehydroalanine followed by thiol nucleophile addition, is suitable for the site-specific installation of a wide range of chemical mimics of PTMs, including acetylated and dimethylated lysine, and other unnatural amino acids. These reactions, optimized for the clinically relevant kinase Aurora-A, readily proceed to completion as revealed by intact protein mass spectrometry. Moreover, these reactions proceed under non-denaturing conditions, which is desirable when working with large protein substrates. We have determined reactivity trends for a diverse range of thiol nucleophile addition reactions at two separate sites on Aurora-A, and we also highlight limitations when using thiol nucleophiles that contain basic functional groups. We show that chemical modification of cysteine residues is possible not only on a flexible surface-exposed loop, but also within a deep active site pocket at the conserved DFG motif, which reveals the potential use of this method in exploring enzyme function through modification of catalytic site residues.

## Introduction

Post-translational modifications (PTMs) play a crucial role in the function and regulation of numerous proteins [1], and homogeneous samples of recombinant proteins selectively modified with a desired PTM are indispensible when studying the functions or effects of such modifications. Consequently, the development and application of methods that enable specific and complete modification of selected protein residues under native conditions is essential, and chemical approaches are rapidly emerging as a way to tackle this challenge.

In the past, synthetic mimetics of PTM-modified residues have been incorporated into recombinant proteins by two main techniques: 1) expressed protein ligation [2] and 2) nonsense codon suppression [3]. In the first of these examples, a peptide corresponding to either the N- or C-terminus of the protein, and containing the desired PTM, is synthesized. This is then ligated to the remainder of the protein, which has been produced by standard recombinant protein expression. A major disadvantage of this method is that the desired PTM must be located near a protein terminus, which limits the opportunity for modification in larger proteins. A second disadvantage is the difficulty in achieving overexpression of the target protein lacking part of its N- or C-terminus. The second example, nonsense codon suppression, utilizes non-coding codons and modified tRNAs to incorporate desired PTM-modified residues during recombinant protein production. However, the resulting PTM-proteins are generally recovered in poor yield. Furthermore, this technique is dependent on the ability of synthetic PTM amino acids to permeate the cell membrane; consequently this method is unsuitable for the incorporation of phosphorylated PTMs. However, these and other related methods can be used to selectively introduce unnatural amino acid residues for bioconjugation applications [4].

A relatively new approach to selectively introduce desired PTMs is through *in vitro* chemical modification of cysteine residues. Recombinant proteins that have been engineered to contain a cysteine residue at a selected site are subjected to synthetic procedures that result in site-specific incorporation of a structural and/or functional mimic of a desired PTM. This approach has been used to generate proteins containing a wide variety of PTMs, such as phosphorylation, glycosylation, methylation, acetylation, and lipidation, which has enabled structural and functional studies that would not otherwise have been feasible [5]–[11]. The modification of a cysteine residue to incorporate a desired modification can be achieved using a number of methods, for example by elimination to dehydroalanine followed by Michael addition of a thiol nucleophile or cross-metathesis; alkylation via a thiol-ene radical-mediated ‘click’ reaction; or by electrophilic or maleimide alkylation of cysteine [12]. The advantage of selective chemical modification approaches over expressed protein ligation or nonsense codon suppression is that desired modifications, including phosphorylation, can be installed anywhere on the protein that is solvent-accessible. Furthermore, these techniques can be used to attach tags such as fluorescent labels [13], ubiquitin [14], or solubilizing groups [15] to specific protein sites. In addition, related strategies exist for the chemical modification of other canonical residues including lysine, tyrosine, tryptophan, glutamate, aspartate, and histidine [16], [17].

**Figure 1:**
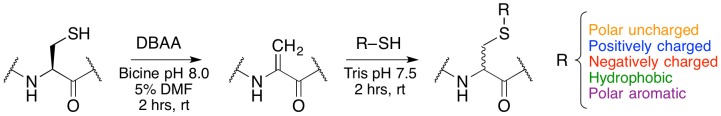
Chemical conversion of cysteine to a range of unnatural amino acid residues.

**Figure 2:**
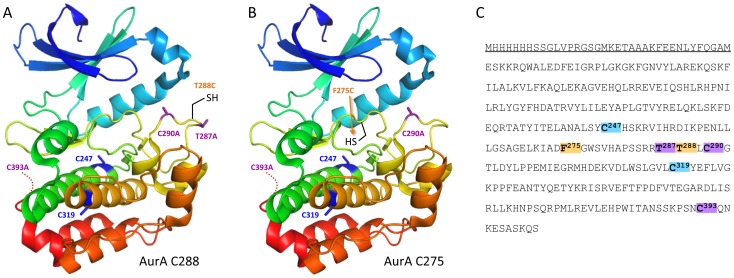
AurA C288 and AurA C275 constructs and protein sequence. (A) AurA C288 contains mutations T287A, T288C, C290A & C393A. (B) AurA C275 contains mutations F275C, C290A & C393A. (C) Sequence of AurA 122–403 kinase domain with *N*-terminal His-tag (underlined). Sites of chemical modification are labelled orange, other mutations are purple, and remaining internal cysteine residues are blue.

Many of the existing literature examples of PTM-incorporation via cysteine functionalization have been carried out on simple, small (<15 kDa) proteins, and often under denaturing conditions. However, using an optimized version of the “tag and modify” approach developed by Chalker and co-workers [6], we have previously incorporated functional phospho-serine mimics at distinct sites on the 37 kDa protein kinase domain of a clinically relevant kinase, Aurora-A (AurA) [18]. Furthermore, the reaction conditions had no detrimental effect on the protein's catalytic activity, or, by extension, its structure. This approach has now been applied to other kinases to study the effect of phosphorylation at different sites [19]. Here, we apply our optimized methodology, using AurA as a relevant model protein, to extend the range of PTMs and other modifications that can be incorporated by this method. We have previously shown that unnatural synthetically generated amino acid residues can act as canonical serine surrogates [18], and we demonstrate here the installation of a diverse array of unnatural residues with varying physico-chemical properties (Figure 1). In addition, we demonstrate that selective cysteine functionalization is possible not only on a flexible surface-exposed loop, at T288C, but is also surprisingly facile on a cysteine residue buried deep within the ATP binding site of AurA, F275C (Figure 2). F275 is within the ‘DFG’ region of the active site, a highly conserved motif that contains key catalytic residues essential for substrate phosphorylation. This motif is known to be flexible in AurA and adopt ‘DFG-in’, ‘DFG-out’ and ‘DFG-up’ conformations [20]. Certain mutations to this conserved region are able to induce stable alternative DFG conformations in p38α MAP Kinase [21], hence we wanted to examine the feasibility of efficiently introducing a variety of non-natural mutations at this site on AurA. Selective chemical modification of a cysteine residue inserted at the DFG+2 position of both p38α and cSrc kinases has been successfully employed to attach an environmentally-sensitive fluorophore [22], [23], which demonstrates that chemical modification near to the DFG motif is indeed feasible; however, based upon observed crystal structures [24], F275 in AurA appears less accessible than the DFG+2 position. In this work we demonstrate the scope of cysteine modification at both a flexible surface-exposed protein loop, and within the buried ATP binding site of AurA. We show that selective cysteine functionalization is a valuable tool with which to mimic post-translational modifications and introduce diverse unnatural residues under native conditions.

## Results and Discussion

### Chemical modification of surface and buried sites

The two constructs of AurA used for this work (T288C and F275C) contain a number of mutations introduced by site-directed mutagenesis (Figure 2). As described previously [18], both constructs contain C290A and C393A mutations to prevent undesired chemical modification from occurring at exposed cysteine residues. Two cysteine residues (C247 and C319) remain; however, our previous work confirmed that these residues are buried within the protein and not accessible to chemical modification. The T288C construct has an additional alanine substitution at the adjacent T287 phosphorylation site because this construct was originally designed to investigate the effect of AurA phosphorylation exclusively at position 288 [18].

**Figure 3:**
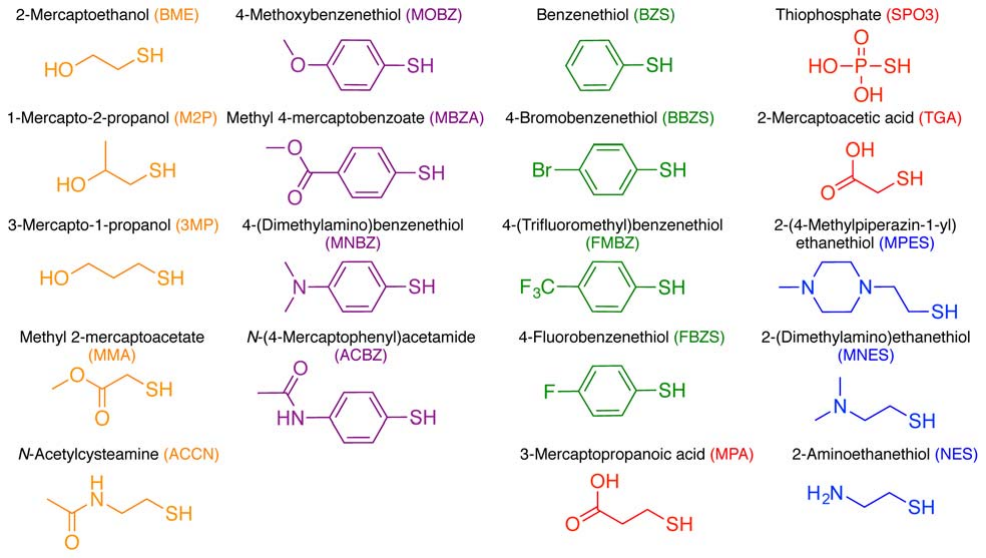
Reagents used for addition at C288^Dha^ and C275^Dha^. Reagents are colored as indicated in Figure 1.

Under identical reaction conditions, C288 and C275 both react to form dehydroalanine (Dha) from cysteine (Figure S1). Formation of C288^Dha^ proceeds to completion within 2 hours consistent with the location of C288 on a flexible surface-exposed loop; however reaction at C275, which is located deep in the kinase ATP binding pocket, does not proceed to completion in 2 hours, resulting in a small amount of residual cysteine at this position which is carried through to the second step of the reaction. We then reacted a wide range of thiol nucleophiles with C288^Dha^ and C275^Dha^ (Figure 3 and Table 1) and determined the success of each reaction by intact protein mass spectrometry (LC-MS) (Figures S2–S3). Consistent with previous reports [18], PTMs corresponding to lysine and *N*-terminal methionine gluconoylation or phospho-gluconoylation were observed resulting in additional peaks of +178 Da or +258 Da alongside that of the expected mass. Pleasingly, we observed no difference in the success of thiol addition reactions at the C275 site compared to the surface exposed C288 site, with the notable exception of the small amount of unreacted C275 cysteine that is carried through each C275 reaction (Table 1). We propose that the high level of modification achieved at C275 highlights the flexibility and accessibility of this region in the absence of a bound ligand, and suggests that in the apo form of AurA, both C275 and C275^Dha^ can adopt a chemically-accessible ‘DFG-out’ conformation in which the side-chain of cysteine and the sp^2^ carbon of dehydroalanine point outwards into the ATP binding site. This is in contrast to published crystal structures that show an inaccessible ‘DFG-in’ conformation of F275 when bound to ATP [24]. Indeed, the modification of F275 to either cysteine or dehydroalanine may make a ‘DFG-out’ conformation more prevalent, as the favorable interactions of the lipophilic phenylalanine side-chain with the hydrophobic pocket observed in the ‘DFG-in’ conformation are reduced. The success of chemical modification at this residue under non-denaturing conditions differs from other methods in which the protein is either subjected to harsher conditions to gain access to the hindered cysteine [25], or is unfolded using guanidinium hydrochloride [9]–[11], both of which are undesirable when working with larger, more complex proteins such as AurA. Additionally, we have previously shown that the reaction conditions applied here are not detrimental to the catalytic activity of AurA thereby demonstrating proof of protein viability under these conditions [18].

**Table 1:** LC-MS results of chemical modification of Aurora-A C288^Dha^ and C275^Dha^.

		AurA C288^Dha^			AurA C275^Dha^		
No.	R–SH^a^	MW	Result*^b^*	Fig.	MW	Result*^b^* ^,^ d	Fig.
**1**	BME	36603	Excellent conversion	S2.A	36587	Excellent conversion	S3.A
**2**	M2P	36617	Very good conversion, tiny amount Dha remains	S2.B	36601	Good conversion, some Dha remains	S3.B
**3**	3MP	36617	Very good conversion, tiny amount Dha remains	S2.C	36601	Good conversion, some Dha remains	S3.C
**4**	MMA	36631	Excellent conversion	S2.D	36615	Excellent conversion	S3.D
**5**	ACCN	36644	Excellent conversion	S2.E	36628	Excellent conversion (1500 eq.)	S3.E
**6**	MOBZ	36665	Excellent conversion	S2.F	36649	Excellent conversion (1500 eq.)	S3.F
**7**	MBZA	36693	Excellent conversion	S2.G	36677	Excellent conversion	S3.G
**8**	MNBZ	36678	Excellent conversion	S2.H	36662	Excellent conversion (1500 eq.)	S3.H
**9**	ACBZ	36692	Excellent conversion	S2.I	36676	Excellent conversion	S3.I
**10**	BZS	36635	Excellent conversion	S2.J	36619	Excellent conversion (1500 eq.)	S3.J
**11**	BBZS	36714	Excellent conversion	S2.K	36698	Excellent conversion (1500 eq.)	S3.K
**12**	FMBZ	36703	Excellent conversion	S2.L	36687	Excellent conversion (1500 eq.)	S3.L
**13**	FBZS	36653	No reaction, still Dha	S2.M	36637	No reaction, still Dha	S3.M
**14**	MPA	36631	Dha, unidentified peaks, some correct mass	S2.N	36615	Dha, unidentified peaks, tiny amount correct mass	S3.N
**15**	SPO3	36639	Good conversion, some Dha remains	S2.O	36623	n/d^e^	–
**16**	TGA	36617	Dha, additional unidentified peak	S2.P	36601	Dha, additional unidentified peaks	S3.O
**17**	MPES	36685	No reaction, Dha remains	S2.Q	36669	Tiny amount correct mass, mostly still Dha	S3.P
**18**	MNES	36630	Excellent conversion, also 102 Da adduct	S2.R	36614	Excellent conversion, also 103 Da adduct	S3.Q
**19**	NES	36602	n/d^c^	–	36586	Multiple unidentified peaks (1500 eq.)	S3.R

a Compounds grouped by properties: polar uncharged (**1–5**), polar aromatic (**6–9**), hydrophobic (**10–13**), negatively charged (**14–16**), and positively charged (**17–19**). *^b^*Reaction of Dha with 5000 eq. of thiol reagent, unless otherwise stated.

c Not determined, likely to denature protein.

d C275 cysteine to Dha reaction is incomplete, so unreacted cysteine is present in all final products.

e Not determined for C275; results for C288 from previous work [18]. ‘Excellent conversion’ is defined as full reaction of Dha to the desired product, with no peaks observed by LC-MS to indicate unreacted Dha.

We were able to determine general reactivity trends for thiol addition reactions. Polar uncharged and polar aromatic thiols all react efficiently with Dha at both C288 and C275 (Table 1, entries **1–9**). We used 5000 eq. of each thiol reagent as standard conditions, however for one example, 4-methoxybenzenethiol (MOBZ), we determined the minimum excess required to drive full conversion. We found AurA 288^Dha^ reacted completely to the desired MOBZ product when >250 eq. of MOBZ were used; whereas reactions with up to 250 eq. MOBZ still contained unreacted Dha after 2 hours (Figure S4). Hydrophobic aromatic thiols also react to completion under our standard conditions (**10–12**), with the exception of 4-fluorobenzenethiol (FBZS, **13**), which did not react at either site presumably as a result of lower nucleophilicity. Representative examples of different thiol reactions that showed excellent conversion at both sites are provided in the Supporting Information, including LC-MS/MS confirmation of the site of modification and quantification of protein recovery after the reaction, which is typically around 75% (Figure S5).

We observed that thiol addition reactions using charged reagents were less successful than those using uncharged or aromatic thiols (Table 1, **14–19**). Negatively charged reagents either did not react, or did not react to completion (**14–16**) consistent with previous work in which thiophosphate [(SPO_3_)^3−^] addition to Dha at C288 was incomplete and required purification to obtain the desired product [18]. Furthermore, additional unidentified peaks were observed by LC-MS for reactions with 3-mercaptopropanoic acid (MPA, **14**) and 2-mercaptoacetic acid (TGA, **16**). Using positively charged thiol reagents gave mixed results (**17–19**). Reactions using 2-(4-methylpiperazin-1-yl)ethanethiol (MPES, **17**) were unsuccessful whilst reactions with 2-(dimethylamino)ethanethiol (MNES, **18**) worked efficiently to give the desired product, however adducts of 102–103 Da were observed on both C288 and C275 samples (*vide infra*); and reaction of C275 with 2-aminoethanethiol (NES, **19**) resulted in multiple unidentified peaks observed by LC-MS with only a small amount of the C275^Dha^ starting material detected. A thermal denaturation screen, conducted to optimize buffer conditions, revealed that 2-aminoethanol completely unfolds AurA when used as a buffer component at a concentration of >5% (v/v) (Figure S6). It is therefore likely that NES has a similar effect and, although used at a lower concentration, acts to denature the protein.

### Synthesis of *N*-acetyl-lysine and *N*, *N*-dimethyl-lysine mimics

**Figure 4:**
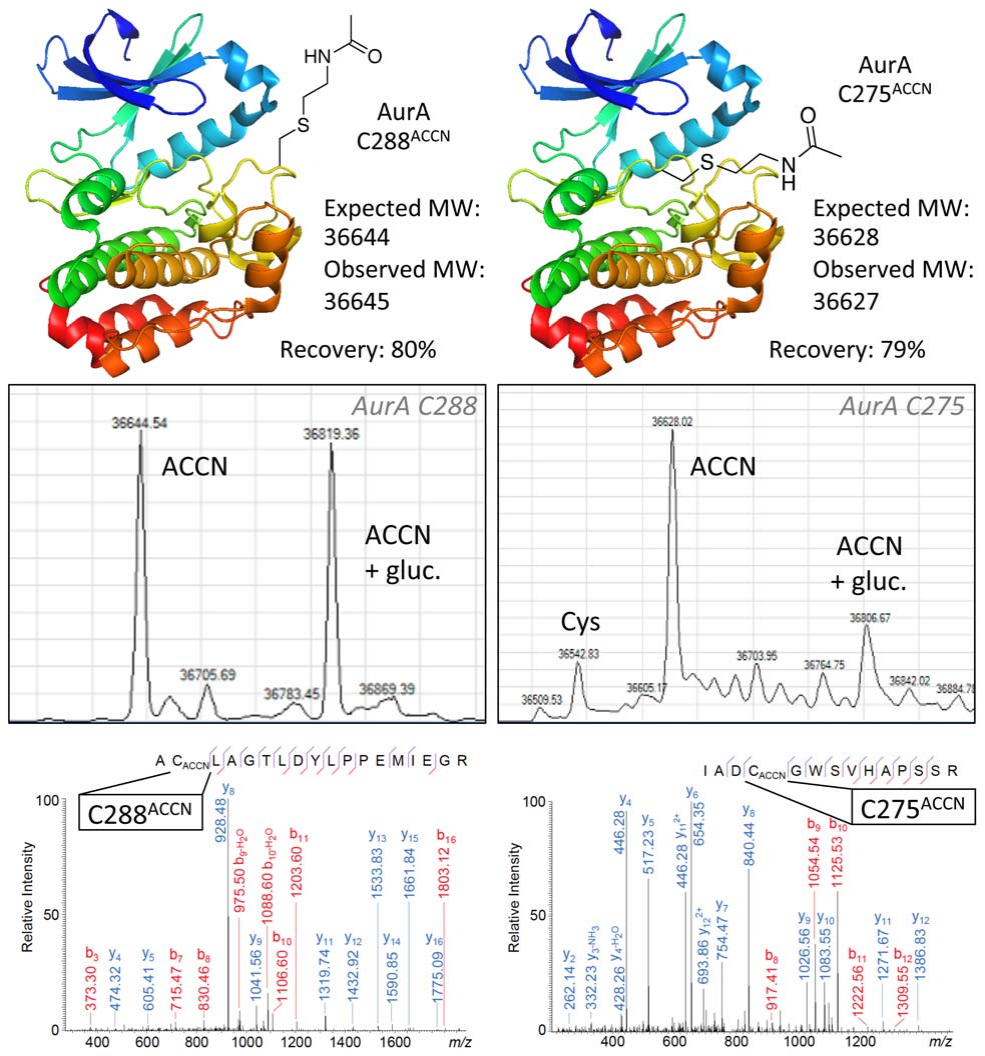
Generation of *N*-acetyl-lysine mimic at exposed and hindered sites on AurA. LC-MS indicates complete conversion of Dha to ACCN product at C288 and C275, with a small amount of unreacted cysteine present at C275. LC-MS/MS confirms the sites of modification.

In addition to generating a range of unnatural amino acids, we produced a chemical mimic of *N*-acetyl-lysine using *N*-acetylcysteamine (ACCN, **5**). This reaction was successful at both the C288 and F275C sites (Figure 4), reacted to completion and yielded a protein recovery of ∼80%. This demonstrates the potential of this method to incorporate mimics of PTM-residues in a site-specific manner for applications where selective lysine *N*-acetylation is desired, such as for *in vitro* studies of protein epigenetic modification and analogous to our previous work describing the selective installation of phospho-serine mimics to facilitate studies of kinase activation [18]. Li and co-workers have described an elegant alternative method to generate an *N*-acetyl-lysine mimic via radical-mediated thiol-ene addition [11], however their substrate proteins were significantly smaller; namely: Ubiquitin (8.5 kDa), Histone H3 (15 kDa) and Histone H4 (11 kDa). Furthermore, their reactions on histones were carried out under denaturing conditions. Additionally Chalker and co-workers previously applied the synthetic method described here to incorporate an *N*-acetyl-lysine mimic into Histone H3 [6]. The work described here extends the methodology to a much larger protein, AurA kinase, and proceeds under native conditions that are more likely to maintain the integrity of larger protein substrates.

**Figure 5:**
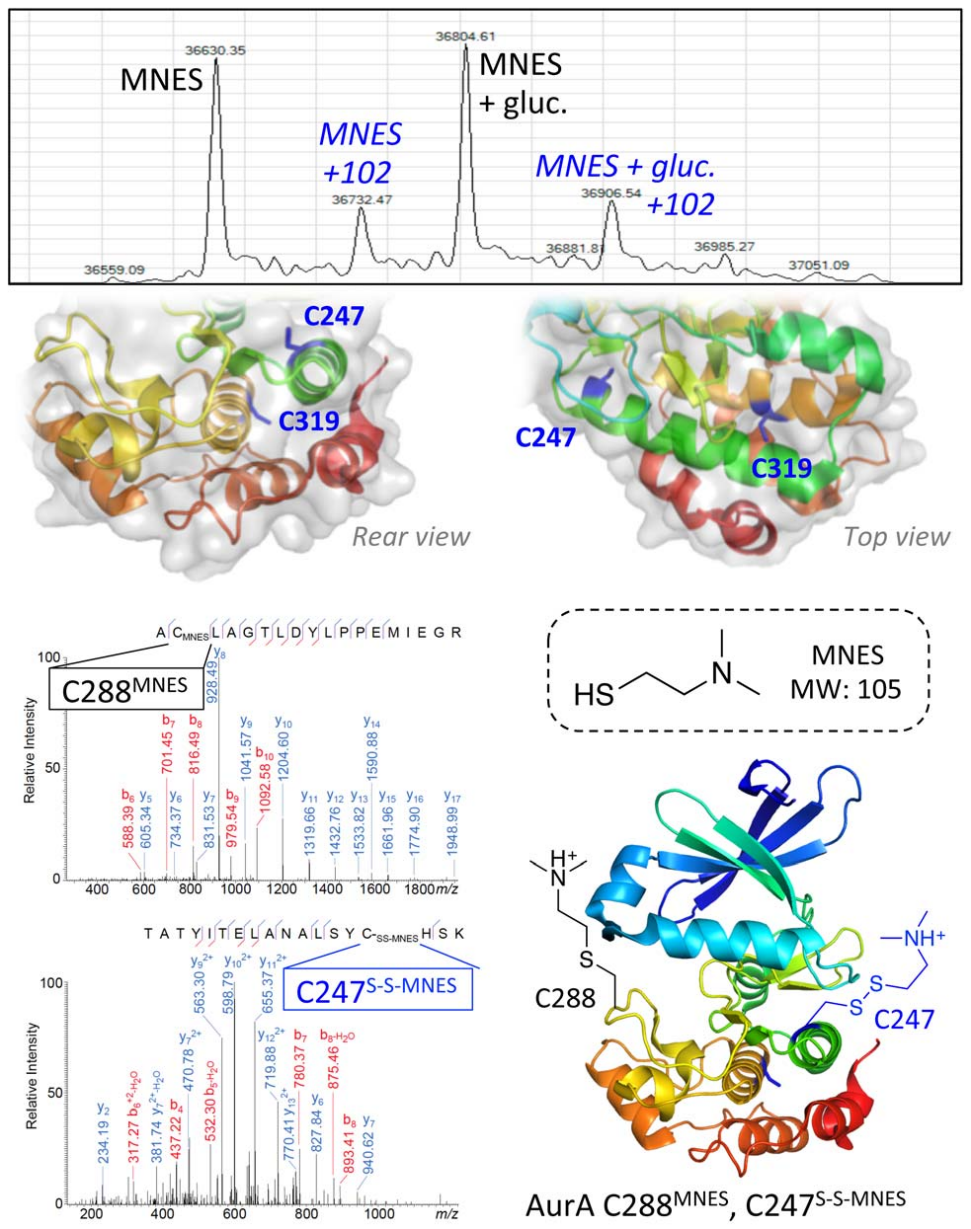
Identification of MNES adducts on AurA. Extra mass adducts of 102 Da were observed on AurA C288 after reaction with MNES in addition to the expected C288 MNES product. This mass corresponds (within the error associated with intact protein LC-MS) to the MNES reagent, which was confirmed by LC-MS/MS as forming a disulfide-linked adduct with C247, which is close to the surface of AurA and could become accessible with slight protein unfolding.

We reacted C288^Dha^ and C275^Dha^ with MNES to specifically generate an *N*, *N*-dimethyl-lysine mimic, a procedure of potential general use in the study of lysine methylation states under epigenetic control. Both C288^Dha^ and C275^Dha^ reacted to completion to give the desired product (**18**), however, extra mass additions of 102–103 Da were also observed on both samples (Figures S2.R and S3.Q). The extra mass was indicative of disulfide-linked adducts of the 105 Da MNES reagent with one of the internal cysteines within AurA (Figure 5). The constructs of AurA used in this study contain two internal buried cysteines, C247 and C319, which were predicted to be inaccessible to chemical modification (Figure 2). However, we postulate that the positively charged MNES reagent causes partial unfolding of AurA to expose C247 and facilitate formation of a disulfide bond with MNES (Figure 5). Chalker and co-workers have previously demonstrated the successful reaction of MNES with Histone H3 to produce an *N*, *N*-dimethyl-lysine mimic [6]; however, in this case, the construct did not contain any bystander cysteines that would be prone to side reaction. An alternative method described by Simon and co-workers for the selective installation of methylated lysine analogues via cysteine alkylation uses denaturing conditions that would be unsuitable for application to larger proteins such as AurA [10]. Although both methods are appropriate for the modification of small protein substrates containing a single cysteine residue, the conditions we demonstrate here are applicable to larger protein substrates with the proviso that caution should be exercised when using positively charged thiol reagents that may have the potential to elicit protein unfolding and potential side reactions at bystander cysteine residues.

## Conclusions

In conclusion, we demonstrate the versatility of a reaction initially developed by Chalker and colleagues by extending the range and type of unnatural amino acids that can be incorporated into a significantly more complex protein substrate, and have shown that chemical modification of cysteine residues is possible not only on a flexible loop but is also surprisingly facile within a deep binding site pocket. The successful chemical modification of a cysteine residue located within the ATP binding site of AurA kinase suggests this method could be applied to explore enzyme function through modification of catalytic site residues. We show reactivity trends for thiol additions to Dha residues, demonstrate examples with high levels of conversion and protein recovery, and highlight limitations with positively charged thiol reagents where partial protein denaturation is observed. In addition, we have highlighted two lysine PTM mimics that can be introduced site-specifically under mild non-denaturing conditions to a protein substrate significantly larger than those previously reported. We anticipate that the methods presented here will facilitate studies in which complete and specific PTM, unnatural amino acid or other thiol nucleophile incorporation into large protein substrates is required.

## Methods

### AurA mutagenesis, expression and purification

Site-directed mutagenesis of AurA kinase domain (122–403) in a pET30-based vector was carried out using a QuikChange protocol (Agilent). The mutations were C290A, C393A, and either F275C for the 275 construct or T288C and T287A for the 288 construct. Proteins were expressed in *E. coli* BL21-CodonPlus -RIL (Agilent) or Rosetta 2 (Merck) DE3 cells in LB medium, with initial growth at 37°C followed by overnight incubation at 21°C after induction with 1 mM isopropyl β-D-1-thiogalactopyranoside. Bacterial pellets were resuspended in lysis buffer (50 mM Tris pH 7.5, 300 mM NaCl, 5 mM MgCl_2_, 10% glycerol, 40 mM imidazole), supplemented with protease inhibitors and DNase (Roche), lysed by sonication and clarified by centrifugation. Lysates were filtered then subjected to Ni^2+^ affinity chromatography (GE Healthcare). Proteins were eluted in lysis buffer containing 250 mM imidazole, purified to homogeneity by S75 size exclusion chromatography (GE Healthcare) (gel filtration buffer: 50 mM Tris pH 7.5, 200 mM NaCl, 5 mM MgCl_2_, 10% glycerol, 10 mM β-mercaptoethanol (BME)), then concentrated to ∼10 mg/mL and flash frozen for future use. Protein concentrations were measured in triplicate with a ND-1000 Spectrophotometer (NanoDrop) using molecular weights and extinction coefficients calculated by ProtParam (ExPASy).

### Chemical modification of AurA C288 and C275

α,α′-Di-bromo-adipoyl(bis)amide (DBAA) was synthesized and characterized as described [26]. An aliquot of protein at ∼10 mg/mL* was shaken for 15 min at room temperature (RT) with 10 mM dithiothreitol then exchanged using a PD MiniTrap G-25 column (GE Healthcare) into 50 mM bicine pH 8.0. A 500× molar excess of DBAA dissolved in dimethylformamide (DMF) was added to the eluted protein (final DMF concentration was 5%; additional bicine buffer was added if necessary), and the reaction was shaken at RT for 2 hours to form the dehydroalanine. Protein was desalted and exchanged into 50 mM Tris pH 7.5 over either a PD MidiTrap G-25 or PD-10 column (GE Healthcare), depending on sample volume. Protein concentrations were typically 0.2–0.5 mg/mL by this stage due to dilution. For thiol addition reactions, 5000 eq. (as standard, although some reactions were carried out using 1500 eq.) of thiol nucleophile reagent (various suppliers) were added to 500 µL aliquots of the desalted protein and shaken for 2 hours at RT. The reaction mixture was either analyzed directly by LC-MS, or cleaned up by exchanging into gel filtration buffer without BME for protein quantitation and LC-MS/MS.

*Typically 12.5–40 µL protein was used for each anticipated thiol addition reaction. 125 µL of protein was used for each reaction where final clean-up and quantitation were intended, in order to maintain high enough protein concentration for accurate quantitation (i.e. >0.5 mg/mL).

### Mass spectrometry

Mass spectrometry methods are available as Protocol S1.

## Supporting Information

Figure S1 **Conversion of cysteine to dehydroalanine on AurA C288 (A) and AurA C275 (B).** Intact protein LC-MS indicates complete conversion on C288, and nearly complete conversion on C275 with a small amount of cysteine left unreacted.
(TIF)

Figure S2 **Reaction of AurA C288^Dha^ with thiol nucleophiles.** Unidentified peaks are indicated by a question mark.
(PDF)

Figure S3 **Reaction of AurA C275^Dha^ with thiol nucleophiles.** Unidentified peaks are indicated by a question mark. Cysteine present in reaction mixtures is due to incomplete cysteine to dehydroalanine conversion.
(PDF)

Figure S4 **Optimization of amount of MOBZ required for full conversion of AurA C288^Dha^.** Dha is completely eliminated after 2 hours when using >250 eq. of MOBZ.
(TIF)

Figure S5 **Examples of thiol nucleophile addition reactions that worked well on AurA C288 (A–C) and AurA C275 (D–F).** LC-MS/MS confirms the location of chemical modifications. The reaction yield is calculated by measuring the total protein recovery of modified AurA (i.e. after Dha and thiol nucleophile reactions and clean-up) and comparing against the amount of starting material, as it is not feasible to accurately quantitate chemical conversion by intact mass spectrometry.
(TIF)

Figure S6 **Thermal denaturation curves of AurA with dimethylformamide and 2-aminoethanol (i.e. potential solvents for DBAA) included as buffer components.** Dimethylformamide is tolerated well by AurA, whereas 2-aminoethanol appears to completely denature the protein when used at concentrations above 5%. n/d =  Not determinable.
(TIF)

Protocol S1 **Mass spectrometry methods.**
(PDF)
