# Supplementary figures and images for: Diverse Functionalization of Aurora-A Kinase at Specified Surface and Buried Sites by Native Chemical Modification
Source: PLoS One. 2014 Aug 5;9(8):e103935. doi: 10.1371/journal.pone.0103935 (PMC4122486; doi:10.1371/journal.pone.0103935)

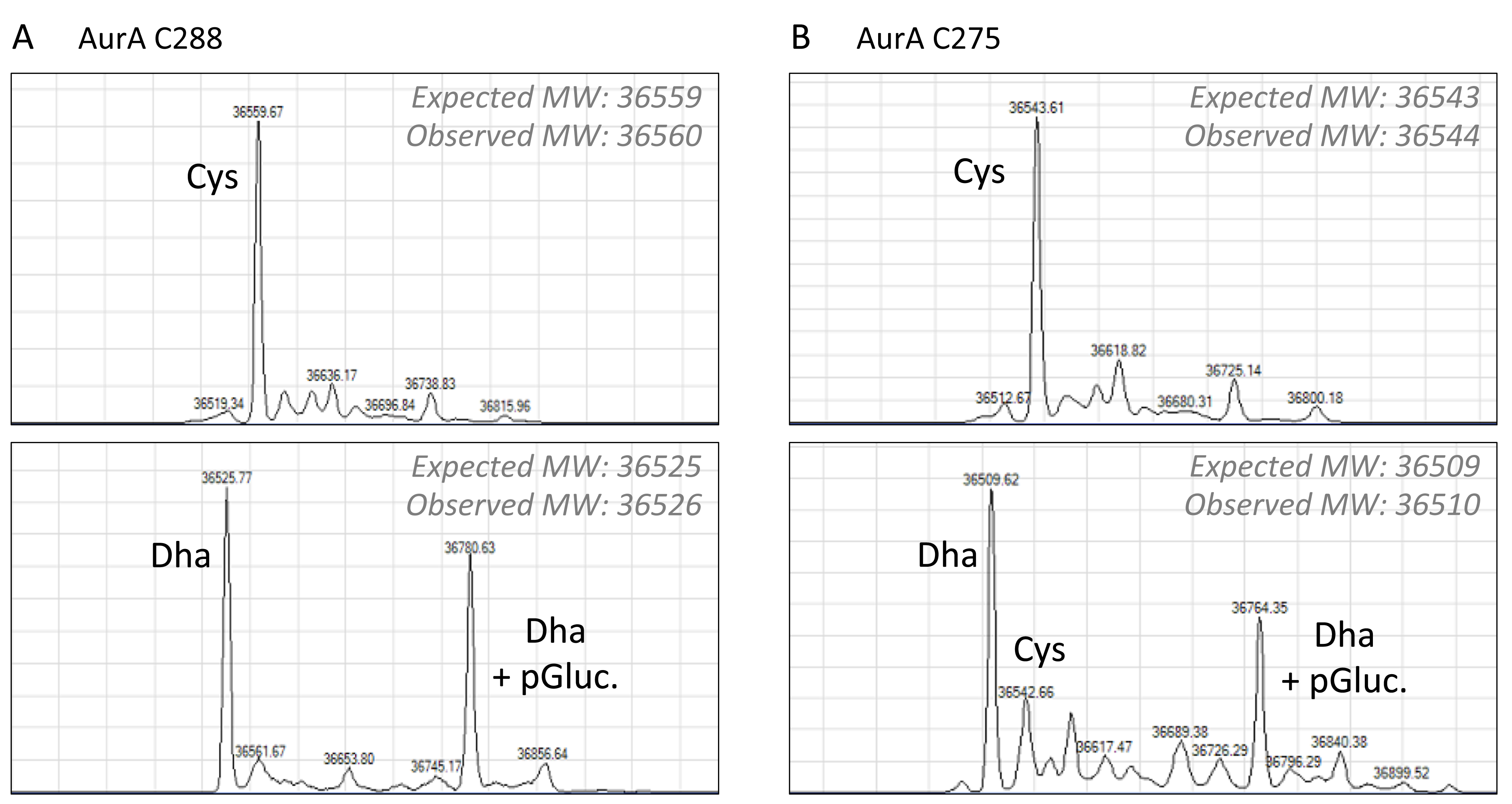

Supplement: Figure S1 — Conversion of cysteine to dehydroalanine on AurA C288 (A) and AurA C275 (B). Intact protein LC-MS indicates complete conversion on C288, and nearly complete conversion on C275 with a small amount of cysteine left unreacted. (TIF) [file pone.0103935.s001.tif]

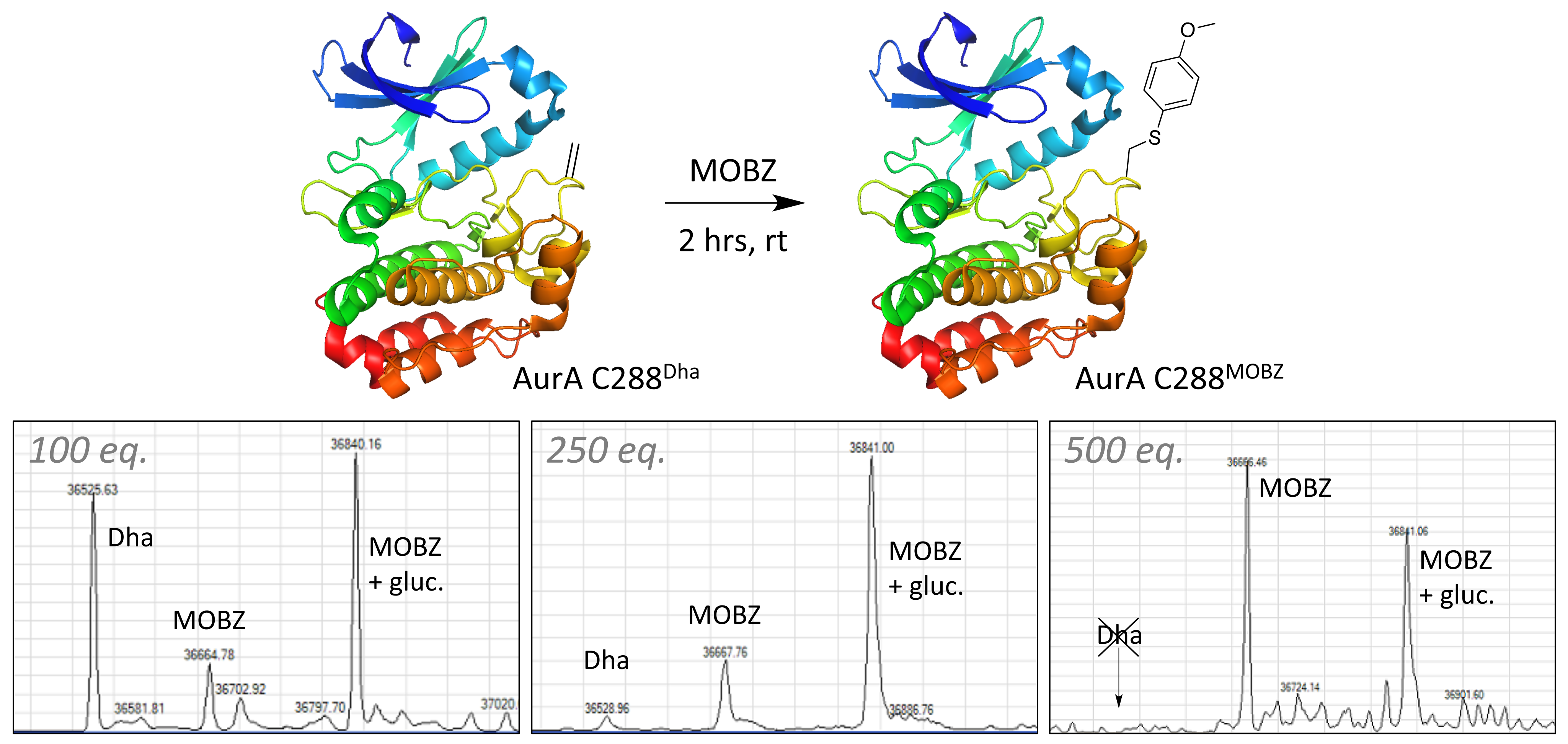

Supplement: Figure S4 — Optimization of amount of MOBZ required for full conversion of AurA C288Dha. Dha is completely eliminated after 2 hours when using >250 eq. of MOBZ. (TIF) [file pone.0103935.s004.tif]

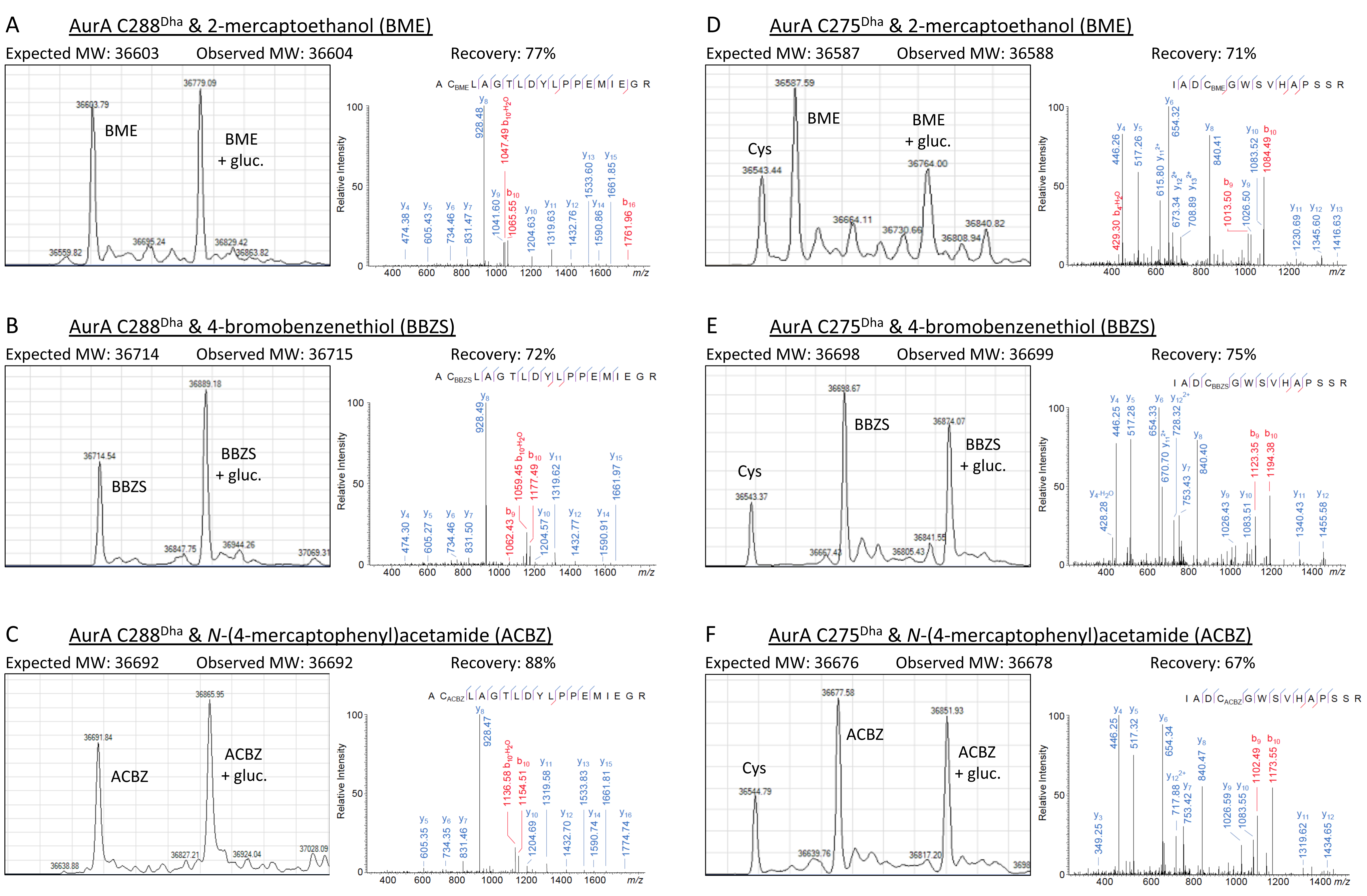

Supplement: Figure S5 — Examples of thiol nucleophile addition reactions that worked well on AurA C288 (A–C) and AurA C275 (D–F). LC-MS/MS confirms the location of chemical modifications. The reaction yield is calculated by measuring the total protein recovery of modified AurA (i.e. after Dha and thiol nucleophile reactions and clean-up) and comparing against the amount of starting material, as it is not feasible to accurately quantitate chemical conversion by intact mass spectrometry. (TIF) [file pone.0103935.s005.tif]

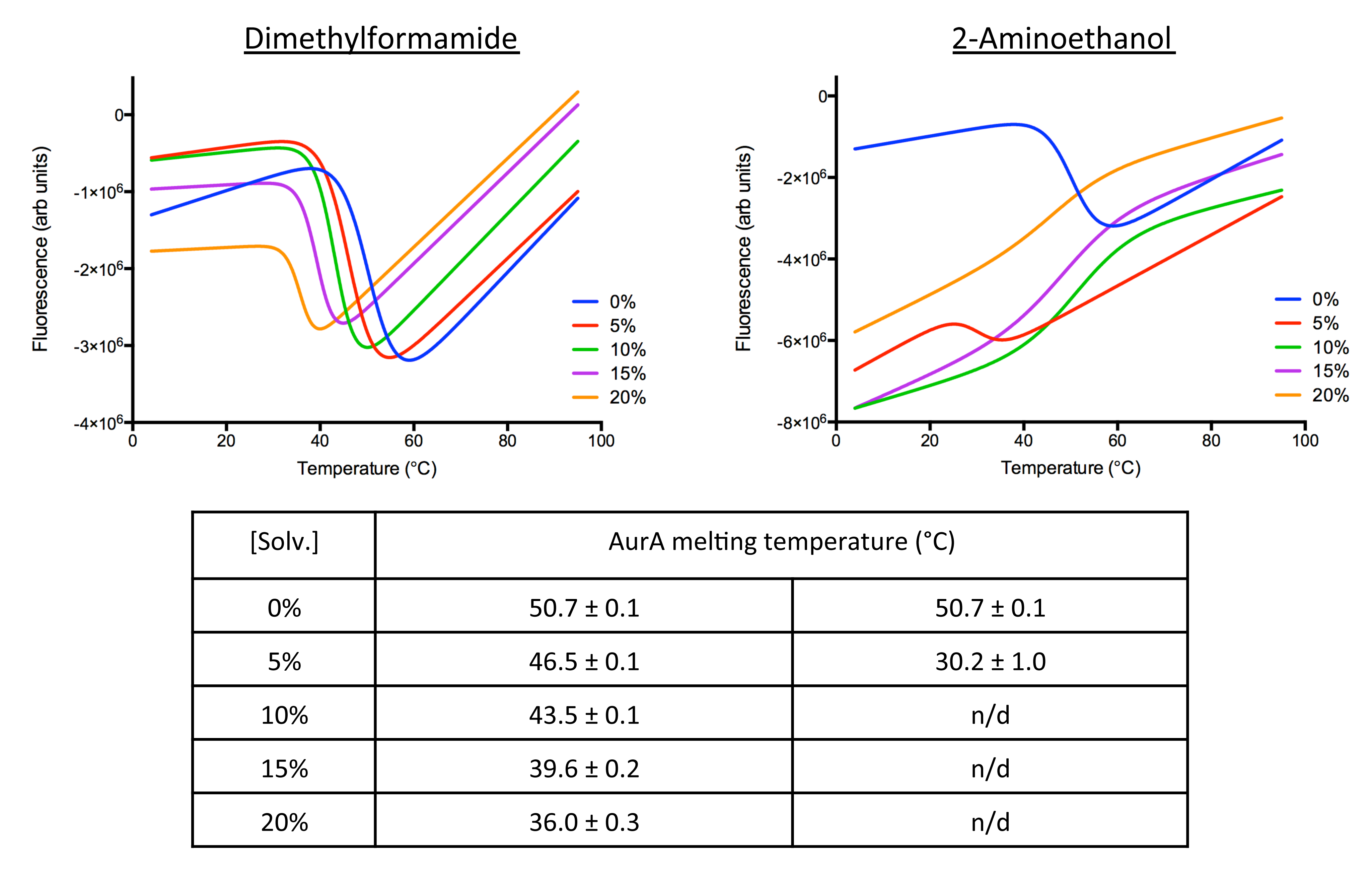

Supplement: Figure S6 — Thermal denaturation curves of AurA with dimethylformamide and 2-aminoethanol (i.e. potential solvents for DBAA) included as buffer components. Dimethylformamide is tolerated well by AurA, whereas 2-aminoethanol appears to completely denature the protein when used at concentrations above 5%. n/d = Not determinable. (TIF) [file pone.0103935.s006.tif]
