# Supplementary material for: Diverse Functionalization of Aurora-A Kinase at Specified Surface and Buried Sites by Native Chemical Modification
Source: PLoS One. 2014 Aug 5;9(8):e103935. doi: 10.1371/journal.pone.0103935 (PMC4122486; doi:10.1371/journal.pone.0103935)

S2.A: AurA C288^{Dha} & 2-mercaptoethanol (BME)

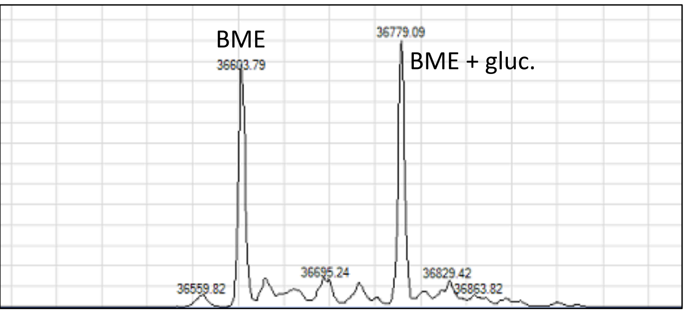

S2.B: AurA C288^{Dha} & 1-mercapto-2-propanol (M2P)

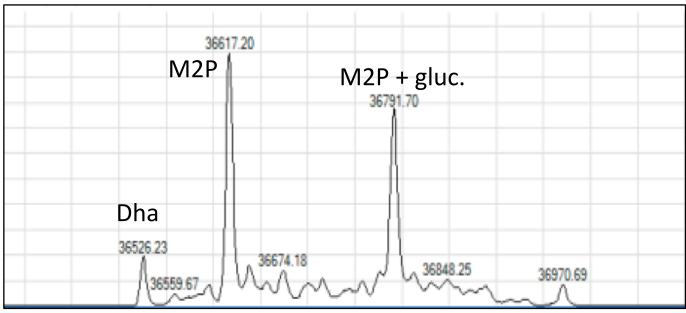

S2.C: AurA C288^{Dha} & 3-mercapto-1-propanol (3MP)

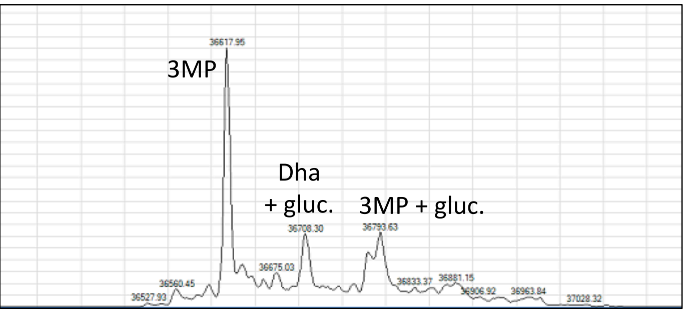

S2.D: AurA C288^{Dha} & methyl 2-mercaptoacetate (MMA)

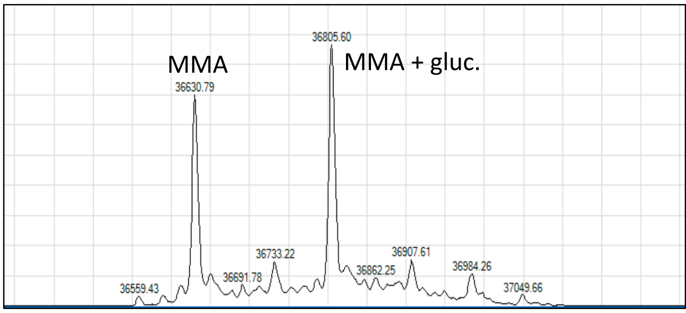

S2.E: AurA C288^{Dha} & *N*-acetylcysteamine (ACCN)

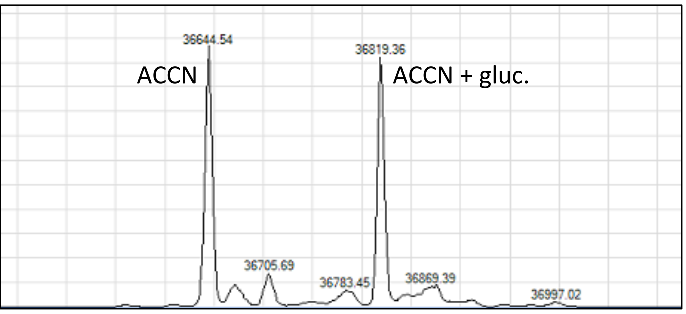

S2.F: AurA C288^{Dha} & 4-methoxybenzenethiol (MOBZ)

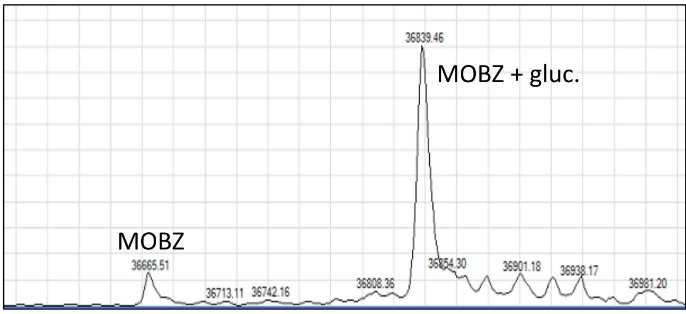

S2.G: AurA C288^{Dha} & methyl 4-mercaptobenzoate (MBZA)

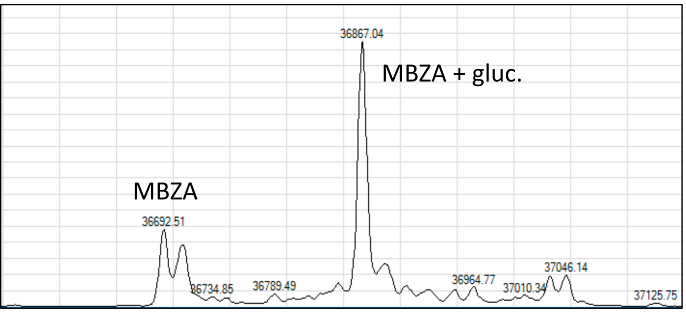

S2.H: AurA C288^{Dha} & 4-(dimethylamino)benzenethiol (MNBZ)

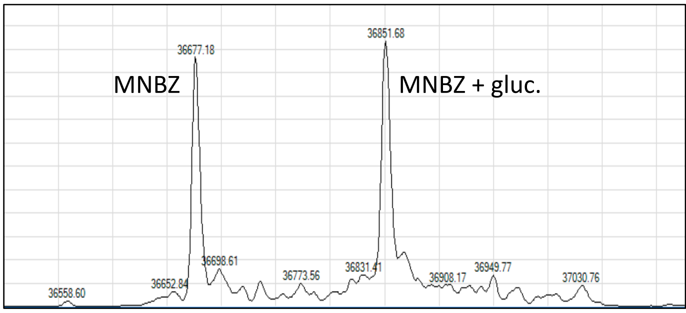

S2.I: AurA C288^{Dha} & *N*-(4-mercaptophenyl)acetamide (ACBZ)

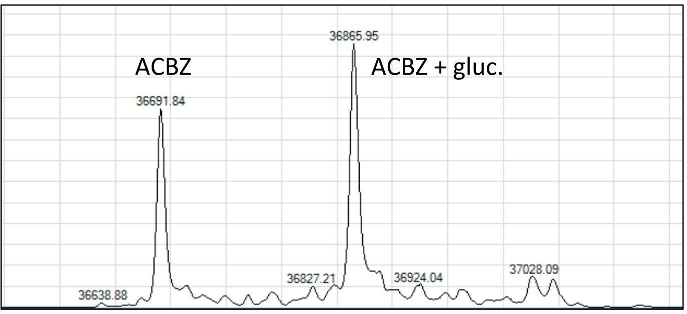

S2.J: AurA C288^{Dha} & benzenethiol (BZS)

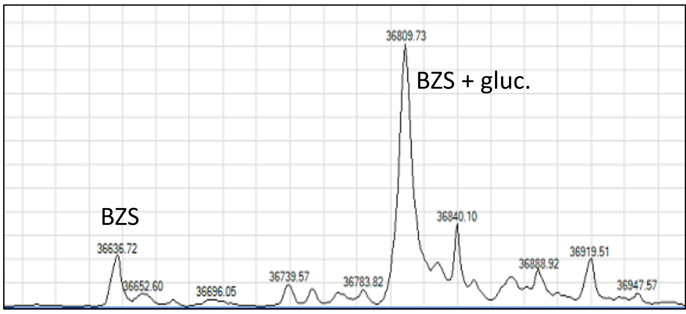

S2.K: AurA C288^{Dha} & 4-bromobenzenethiol (BBZS)

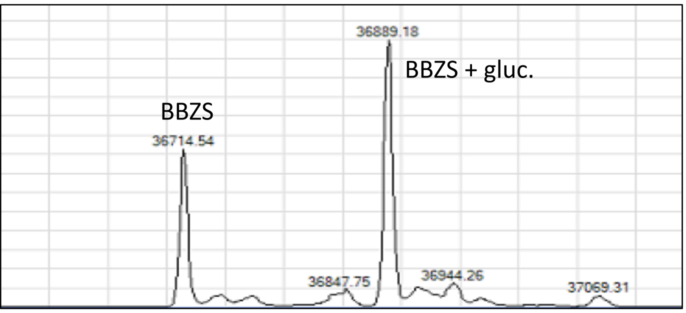

S2.L: AurA C288^{Dha} & 4-(trifluoromethyl)benzenethiol (FMBZ)

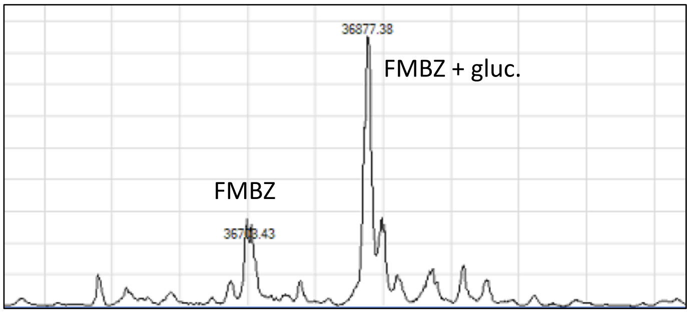

S2.M: AurA C288^{Dha} & 4-fluorobenzenethiol (FBZS)

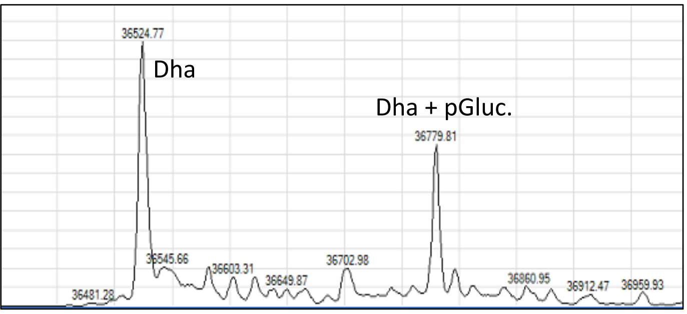

S2.N: AurA C288^{Dha} & 3-mercaptopropanoic acid (MPA)

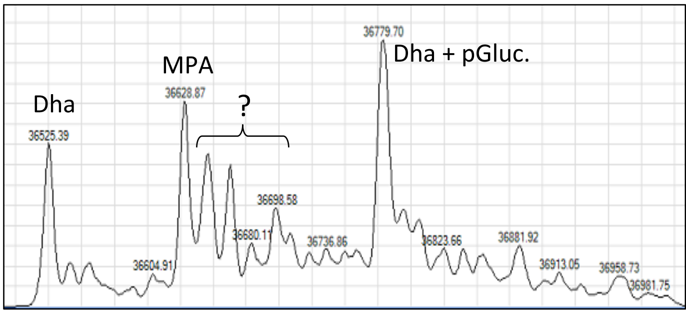

S2.O: AurA C288^{Dha} & thiophosphate (SPO3)

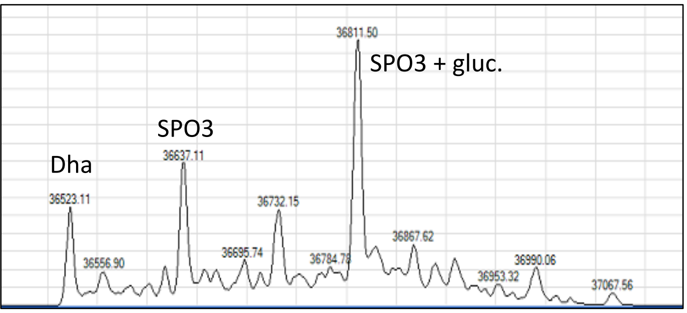

S2.P: AurA C288^{Dha} & 2-mercaptoacetic acid (TGA)

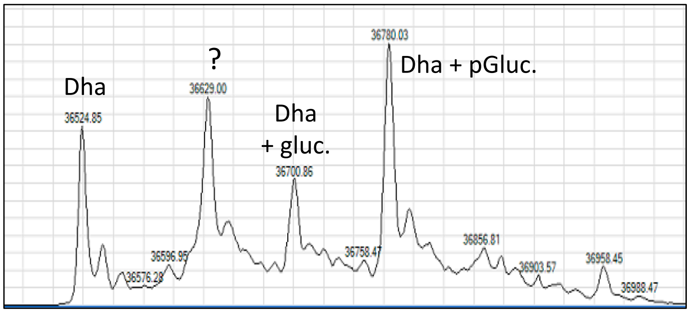

S2.Q: AurA C288^{Dha} & 2-(4-methylpiperazin-1-yl)ethanethiol (MPES)

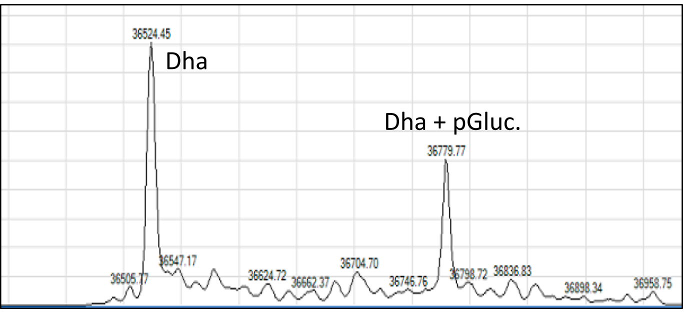

S2.R: AurA C288^{Dha} & 2-(dimethylamino)ethanethiol (MNES)

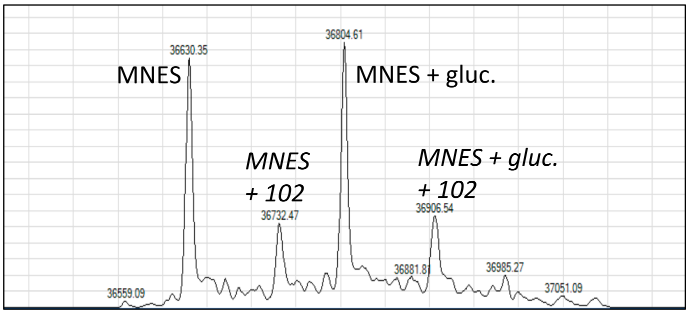

Supplement: Figure S2 — Reaction of AurA C288Dha with thiol nucleophiles. Unidentified peaks are indicated by a question mark. (PDF) [file pone.0103935.s002.pdf]
