# Supplementary material for: Diverse Functionalization of Aurora-A Kinase at Specified Surface and Buried Sites by Native Chemical Modification
Source: PLoS One. 2014 Aug 5;9(8):e103935. doi: 10.1371/journal.pone.0103935 (PMC4122486; doi:10.1371/journal.pone.0103935)

S3.A: AurA C275^{Dha} & 2-mercaptoethanol (BME)

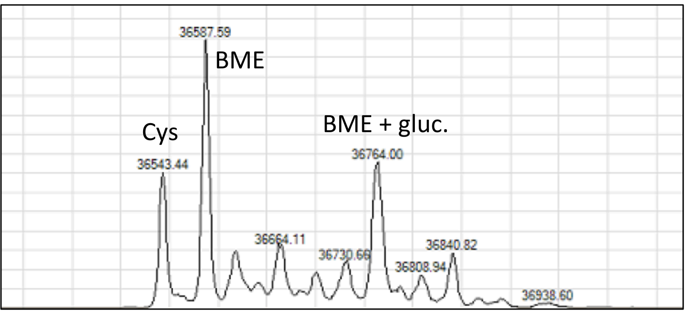

S3.B: AurA C275^{Dha} & 1-mercapto-2-propanol (M2P)

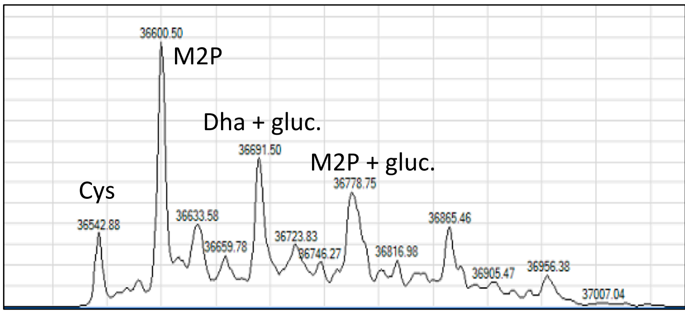

S3.C: AurA C275^{Dha} & 3-mercapto-1-propanol (3MP)

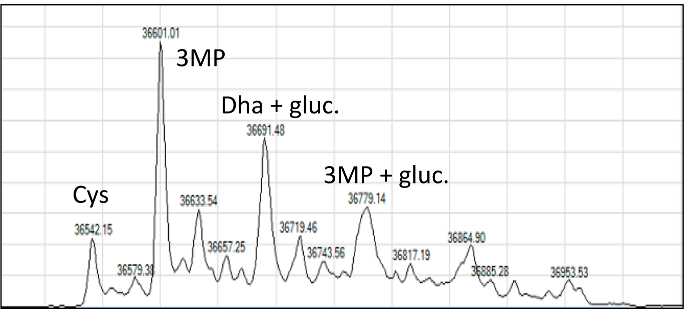

S3.D: AurA C275^{Dha} & methyl 2-mercaptoacetate (MMA)

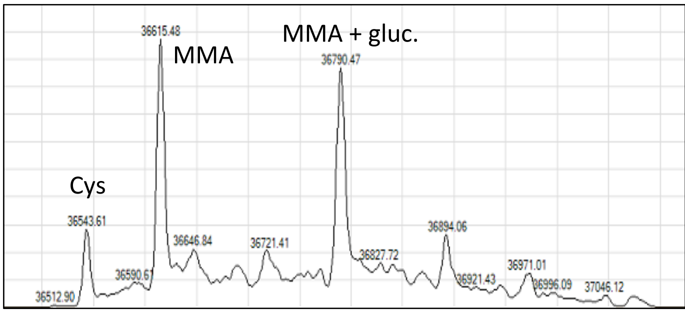

S3.E: AurA C275^{Dha} & *N*-acetylcysteamine (ACCN)

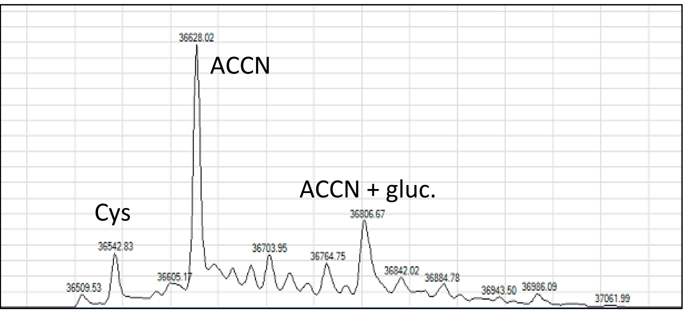

S3.F: AurA C275^{Dha} & 4-methoxybenzenethiol (MOBZ)

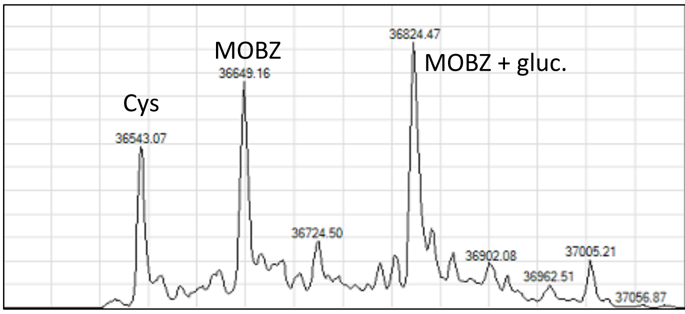

S3.G: AurA C275^{Dha} & methyl 4-mercaptobenzoate (MBZA)

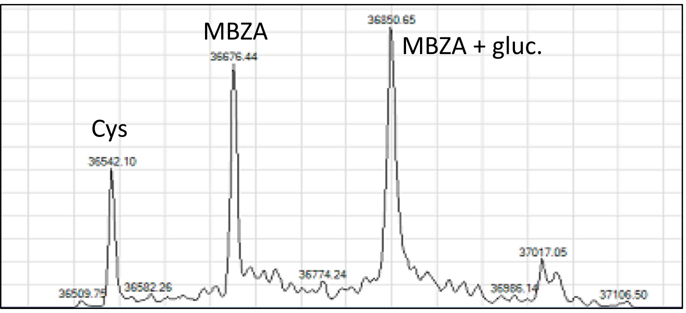

S3.H: AurA C275^{Dha} & 4-(dimethylamino)benzenethiol (MNBZ)

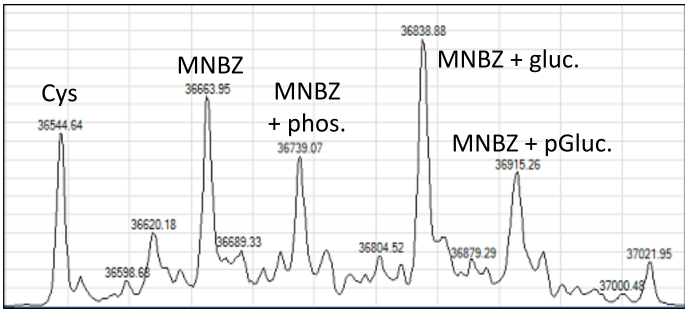

S3.I: AurA C275^{Dha} & *N*-(4-mercaptophenyl)acetamide (ACBZ)

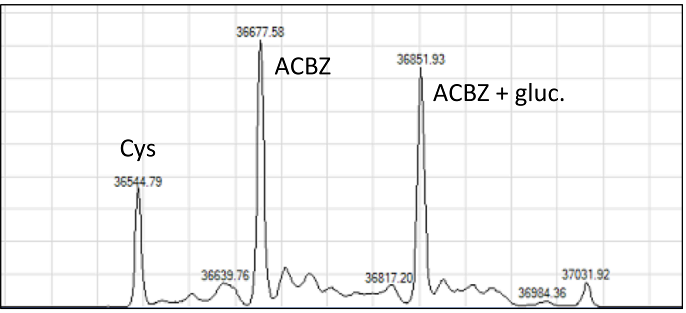

S3.J: AurA C275^{Dha} & benzenethiol (BZS)

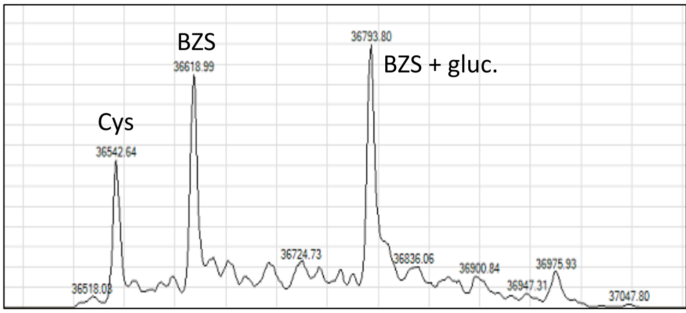

S3.K: AurA C275^{Dha} & 4-bromobenzenethiol (BBZS)

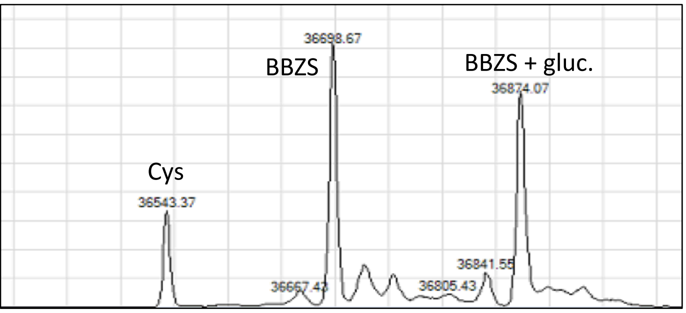

S3.L: AurA C275^{Dha} & 4-(trifluoromethyl)benzenethiol (FMBZ)

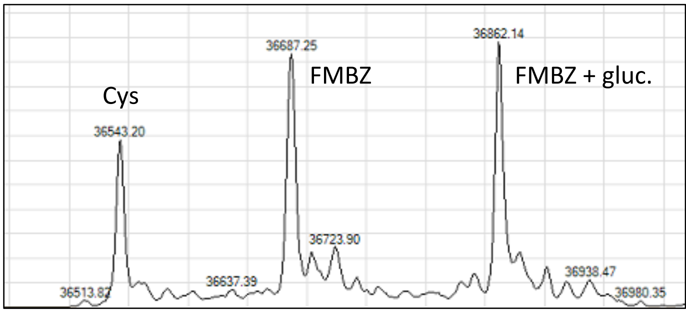

S3.M: AurA C275^{Dha} & 4-fluorobenzenethiol (FBZS)

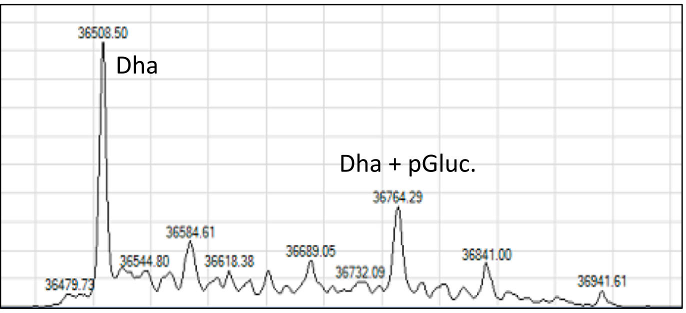

S3.N: AurA C275^{Dha} & 3-mercaptopropanoic acid (MPA)

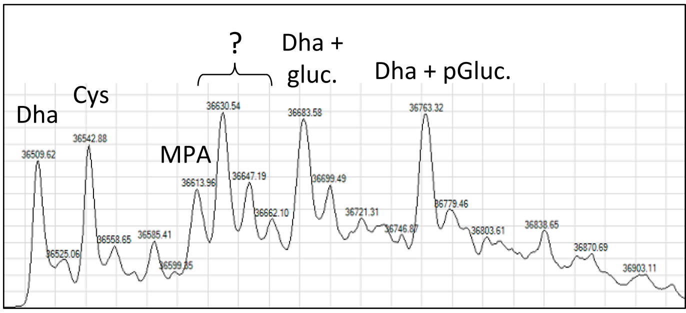

S3.O: AurA C275^{Dha} & 2-mercaptoacetic acid (TGA)

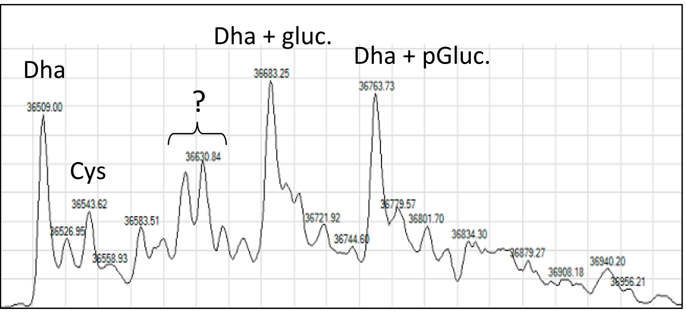

S3.P: AurA C275^{Dha} & 2-(4-methylpiperazin-1-yl)ethanethiol (MPES)

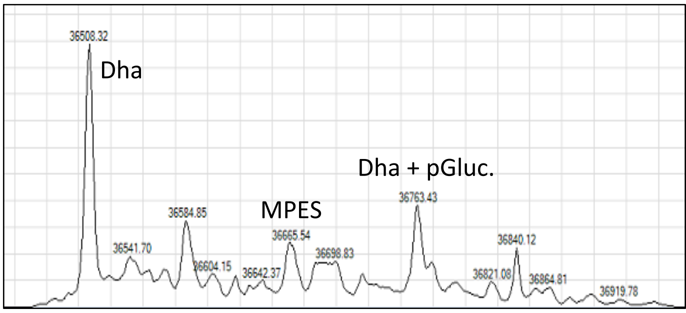

S3.Q: AurA C275^{Dha} & 2-(dimethylamino)ethanethiol (MNES)

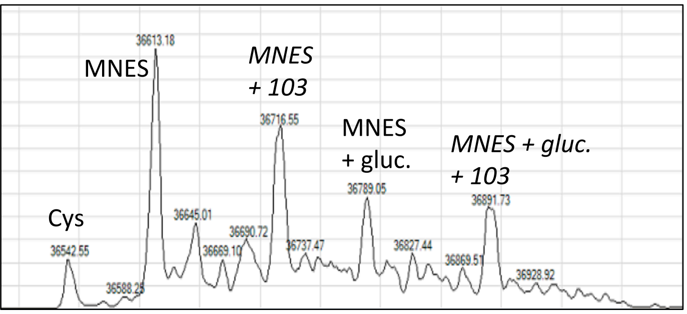

S3.R: AurA C275^{Dha} & 2-aminoethanethiol (NES)

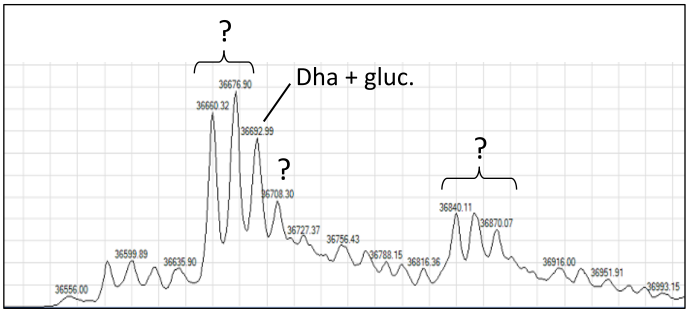

Supplement: Figure S3 — Reaction of AurA C275Dha with thiol nucleophiles. Unidentified peaks are indicated by a question mark. Cysteine present in reaction mixtures is due to incomplete cysteine to dehydroalanine conversion. (PDF) [file pone.0103935.s003.pdf]
