# Supplementary material for: Diverse Functionalization of Aurora-A Kinase at Specified Surface and Buried Sites by Native Chemical Modification
Source: PLoS One. 2014 Aug 5;9(8):e103935. doi: 10.1371/journal.pone.0103935 (PMC4122486; doi:10.1371/journal.pone.0103935)
Supplement: Protocol S1 — Mass spectrometry methods. (PDF) [file pone.0103935.s007.pdf]

Mass Spectrometry Methods

LC-MS for intact proteins

1 µl injections of the sample were made onto a Security Guard C8 column cartridge (4 x 3 mm, AJO-4290, Phenomenex). Samples were refrigerated at 4°C in a G1367B auto-sampler (Agilent) with G1330B thermostat module prior to injection. Chromatographic separation at 60°C was carried out using an Agilent 1200 Series HPLC over a 1 minute gradient elution of 95:5 to 5:95 water and acetonitrile (both modified with 0.1% formic acid) at a flow rate of 0.5 ml/min. The post column eluent flow was infused into an Agilent 6520 Series qToF mass spectrometer fitted with a dual ESI ionization source. An initial divert to waste was used to aid desalting. LC eluent and nebulizing gas was introduced into the grounded nebulizer with spray direction orthogonal to the capillary axis. The aerosol was dried by heated gas (10 L/min of nitrogen at 350°C, 50 psi), producing ions by ESI. Ions entered the transfer capillary along which a potential difference of 4kV was applied. The fragmentor voltage was set at 190V and skimmer at 65V. Signal was optimized by AutoTune.m. Profile mass spectrometry data were acquired in positive ionization mode over a scan range of m/z 650-2000 (scan rate 1.0) with reference mass correction at m/z 922.009798 hexakis(1H,1H,3H-perfluoropropoxy)phosphazene. Raw data were processed using Agilent MassHunter Qualitative Analysis B.06.00 and MagTran 1.02.[1]

LC-MS/MS for Trypsin-digested proteins

AurA samples in Tris buffer (typically 50 mM Tris pH 7.5, 200 mM NaCl, 5 mM $MgCl_2$, 10% glycerol) were directly digested with trypsin, without prior reduction and alkylation. In all cases, digested samples were purified using ziptips (Millipore) essentially according to manufacturer instructions and the dried eluates reconstituted in 0.1% formic acid for LC-MS/MS analysis. Reversed phase chromatography was performed using an HP1200 platform (Agilent) coupled to an LTQ Velos Orbitrap mass spectrometer (Thermo Fisher Scientific). Peptides were resolved on a 75 µm I.D. 15 cm C18 packed emitter column (3 µm particle size; Nikkyo Technos Co., Ltd.) over 30 mins using a linear gradient of 96:4 to 50:50 buffer A:B (buffer A: 2% acetonitrile/0.1% formic acid; buffer B: 80% acetonitrile/0.1% formic acid)at 250 nl/min. Peptides were ionized by electrospray ionization using 1.8 kV applied immediately pre-column to the packed emitter via a microtee built into the nanospray source. Sample was infused into the mass spectrometer directly from the end of the tapered tip silica column (6-8 µm exit bore). The ion transfer tube was heated to 200°C and the S-lens set to 60%. MS/MS were acquired using data dependent acquisition based on a full 30,000 resolution FT-MS scan scan with preview mode disabled and internal lock mass calibration against the polysiloxane ion at 445.120025 *m/z*. The top 10 most intense ions were fragmented by collision-induced dissociation and analysed using enhanced ion trap scans. Precursor ions with unknown or single charge states were excluded from selection. Automatic gain control was set to 1,000,000 for FT-MS and 30,000 for IT-MS/MS, full FT-MS maximum inject time was 500 ms and normalised collision energy was set to 35% with an activation time of 10 ms. Wideband activation was used to co-fragment precursor ions undergoing neutral loss of up to -20 *m/z* from the parent ion, including loss of water/ammonia. MS/MS

was acquired for selected precursor ions with a single repeat count acquired after 5 s delay followed by dynamic exclusion with a 10 ppm mass window for 10 s based on a maximal exclusion list of 500 entries. Raw MS/MS data were compiled into peaklists using Proteome Discoverer v1.3 (Thermo Fisher Scientific) default parameters. Peaklists were interrogated against a SwissProt 2011_01 *Homo sapiens* subset database (20,282 sequences), customised to include AurA construct sequences, using Mascot v2.3 (www.matrixscience.com) and assuming tryptic enzyme specificity with up to two missed cleavages. A precursor ion tolerance of 5 ppm and fragment ion tolerance of 0.25 Da was applied and the following variable modifications were accounted for depending on the sample in question: acetylation of the protein N-terminus, oxidation of methionine, pyroglutamisation of peptide N-terminal glutamine, conversion of cysteine mutations to dehydroalanine, BME-cysteine, -SS-BME, MNES-cysteine, -SS-MNES-cysteine, diMNES-cysteine, phosphorylation of BME-cysteine, ACCN-cysteine, -SS-ACCN-cysteine, BBZS-cysteine, -SS-BBZS-cysteine, ACBZ-cysteine, and -SS-ACBZ-cysteine. The Mascot peptide and protein identification results were grouped and validated using Scaffold v4.0 (Proteome Software Inc.). Protein identifications were automatically accepted if they contained at least 2 unique peptides assigned with at least 95% confidence by Peptide Prophet.[2] To confirm sequence modifications including mutations and chemical derivatisations, the peptide confidence threshold was reduced to 50% and candidate spectra were visually assessed.

(1) Zhang, Z., and Marshall, A. G. (1998) A universal algorithm for fast and automated charge state deconvolution of electrospray mass-to-charge ratio spectra, *J. Am. Soc. Mass Spectrom. 9*, 225–233.

(2) Keller, A., Nesvizhskii, A.I., Kolker, E., and Aebersold, R. (2002) Empirical statistical model to estimate the accuracy of peptide identifications made by MS/MS and database search, *Anal. Chem. 74,* 5383–5392.
